# Immune biomarkers of treatment failure for a patient with renal cell carcinoma on a Phase I trial of pembrolizumab plus radiotherapy

**DOI:** 10.1186/2051-1426-3-S2-P143

**Published:** 2015-11-04

**Authors:** Gregory Alexander, Joshua Palmer, Madalina Tuluc, Jianqing Lin, D Craig Hooper, Bo Lu

**Affiliations:** 1 Sidney Kimmel Medical College at Thomas Jefferson, Philadelphia, PA, USA; 2 Department of Radiation Oncology, Thomas Jefferson University, Philadelphia, PA, USA; 3 Department of Pathology, Jefferson University Hospital, Philadelphia, PA, USA; 4 Department of Medical Oncology, Thomas Jefferson University, Philadelphia, PA, USA; 5 Thomas Jefferson University, Philadelphia, PA, USA

## Background

Pembrolizumab is an antibody designed against programmed cell-death protein-1 (PD-1), which is expressed on the surface of activated T cells. Tumor cells can upregulate PD-L1, which binds to PD-1 and mediates T cell anergy. By antagonizing the PD-1/PD-L1 binding, pembrolizumab can enhance T cell killing in multiple tumor types. RT can be immunostimulatory, therefore, combination therapy may yield enhanced efficacy. Here, we evaluated the immune biomarkers related to a patient who failed combined treatment with radiation and pembrolizumab.

## Methods

Phase I clinical trial (NCT02318771) designed to investigate the immunomodulatory effects of radiation therapy in combination with pembrolizumab. The study randomizes patients to one of four arms: fractionated versus single fraction radiation and pembrolizumab before or after RT. The study enrolls patients with metastatic lung, renal, head and neck cancers, and melanoma. Peripheral blood was obtained before, during, and after treatment were analyzed using flow cytometry. CT guided biopsies of the tumor before and after radiation treatment was preformed and the biopsy samples were subject to RT-PCR analysis.

## Results

The patient was randomized to fractionated radiation 20 Gy in 5 fractions to an anterior chest wall metastasis. Following RT he received 5 cycles of pembrolizumab. He was found to have disease progression at radiated chest wall lesion as well as non-radiated metastatic sites on subsequent image studies. Flow cytometry analysis of his peripheral lymphocyte substance revealed no change in CD8+ cells from baseline (25%) compared to post-radiation (24%) and post-pembrolizumab (24%). CD4+ cells at baseline, post-RT and post-pembrolizumab were 43%, 42% and 35%. Flow cytometry of peripheral blood post-pembrolizumab revealed high level of PD-1 expression on CD8 cells, with low level expression of PD-1 on CD4 cells. RT-PCR on biopsies taken pre-RT and post-RT revealed no T lymphocyte transcripts.

## Conclusions

We present a patient with metastatic clear cell RCC who experienced rapid disease progression following combination treatment. The patient failed to demonstrate expansion of CD8 lymphocytes, the CD4 cells failed to express PD-1 marker and the tumor biopsy before and after RT failed to demonstrate the presence of T lymphocytes. We hypothesize that the lack of tumor infiltrating lymphocytes may play a role in treatment failure. Alternatively, we hypothesize that the tumor microenvironment is heterogeneous with known non-redundant immune checkpoints, which may have contributed to treatment failure. Currently, we are exploring alternative mechanisms of resistance to pembrolizumab by analyzing immunocheckpoints such as Ox-40, CTLA-4, CD73, Tim3, and Lag3.

## Trial registration

ClinicalTrials.gov identifier NCT02318771.

